# Mn^2+^ coordinates Cap-0-RNA to align substrates for efficient 2′-*O*-methyl transfer by SARS-CoV-2 nsp16

**DOI:** 10.1126/scisignal.abh2071

**Published:** 2021-06-29

**Authors:** George Minasov, Monica Rosas-Lemus, Ludmilla Shuvalova, Nicole L. Inniss, Joseph S. Brunzelle, Courtney M. Daczkowski, Paul Hoover, Andrew D. Mesecar, Karla J. F. Satchell

**Affiliations:** 1 Department of Microbiology-Immunology, Northwestern University, Feinberg School of Medicine, Chicago, IL, 60611, USA; 2 Center for Structural Genomics of Infectious Diseases, Northwestern University, Feinberg School of Medicine, Chicago, IL, 60611, USA; 3 Northwestern Synchrotron Research Center, Life Sciences Collaborative Access Team, Northwestern University, Argonne, IL 60439, USA.; 4 Department of Biochemistry and Department of Biological Sciences, Purdue University, West Lafayette, IN, 47907, USA.

## Abstract

Capping of viral messenger RNAs is essential for efficient translation, for virus replication, and for preventing detection by the host cell innate response system. The SARS-CoV-2 genome encodes the 2′-*O*-methyltransferase nsp16, which, when bound to the coactivator nsp10, uses *S*-adenosylmethionine (SAM) as a donor to transfer a methyl group to the first ribonucleotide of the mRNA in the final step of viral mRNA capping. Here, we provide biochemical and structural evidence that this reaction requires divalent cations, preferably Mn^2+^, and a coronavirus-specific four-residue insert. We determined the X-ray structures of the SARS-CoV-2 2′-*O*-methyltransferase (the nsp16-nsp10 heterodimer) in complex with its reaction substrates, products, and divalent metal cations. These structural snapshots revealed that metal ions and the insert stabilize interactions between the capped RNA and nsp16, resulting in the precise alignment of the ribonucleotides in the active site. Comparison of available structures of 2′-*O*-methyltransferases with capped RNAs from different organisms revealed that the four-residue insert unique to coronavirus nsp16 alters the backbone conformation of the capped RNA in the binding groove, thereby promoting catalysis. This insert is highly conserved across coronaviruses, and its absence in mammalian methyltransferases makes this region a promising site for structure-guided drug design of selective coronavirus inhibitors.

## INTRODUCTION

The recently emerged human pathogen SARS-CoV-2 is a positive-stranded RNA virus responsible for the on-going pandemic of highly transmissible fatal respiratory coronavirus infectious disease (COVID-19), which has caused over three million deaths worldwide (1). Like other coronaviruses, SARS-CoV-2 carries a large RNA genome of ~30 kilobases (2), which is directly translated into two polypeptides, Orf1a and Orf1ab. The polyproteins are subsequently autoprocessed into the non-structural proteins (nsps) that comprise the replication and transcription complex (RTC). The genome also serves as a template for viral RNA transcription to generate nine canonical subgenomic mRNAs that encode the SARS-CoV-2 structural and accessory proteins (3). These mRNAs are generated by the RTC through a discontinuous transcription process and thus have identical 5′-ends with an adenosine ribonucleotide at the first position (A_1_) (4, 5). These mRNAs are further modified by 5′-capping to stabilize, improve translation, and protect the RNA from surveillance by the host innate immune system (6, 7).

**Fig. 1.**
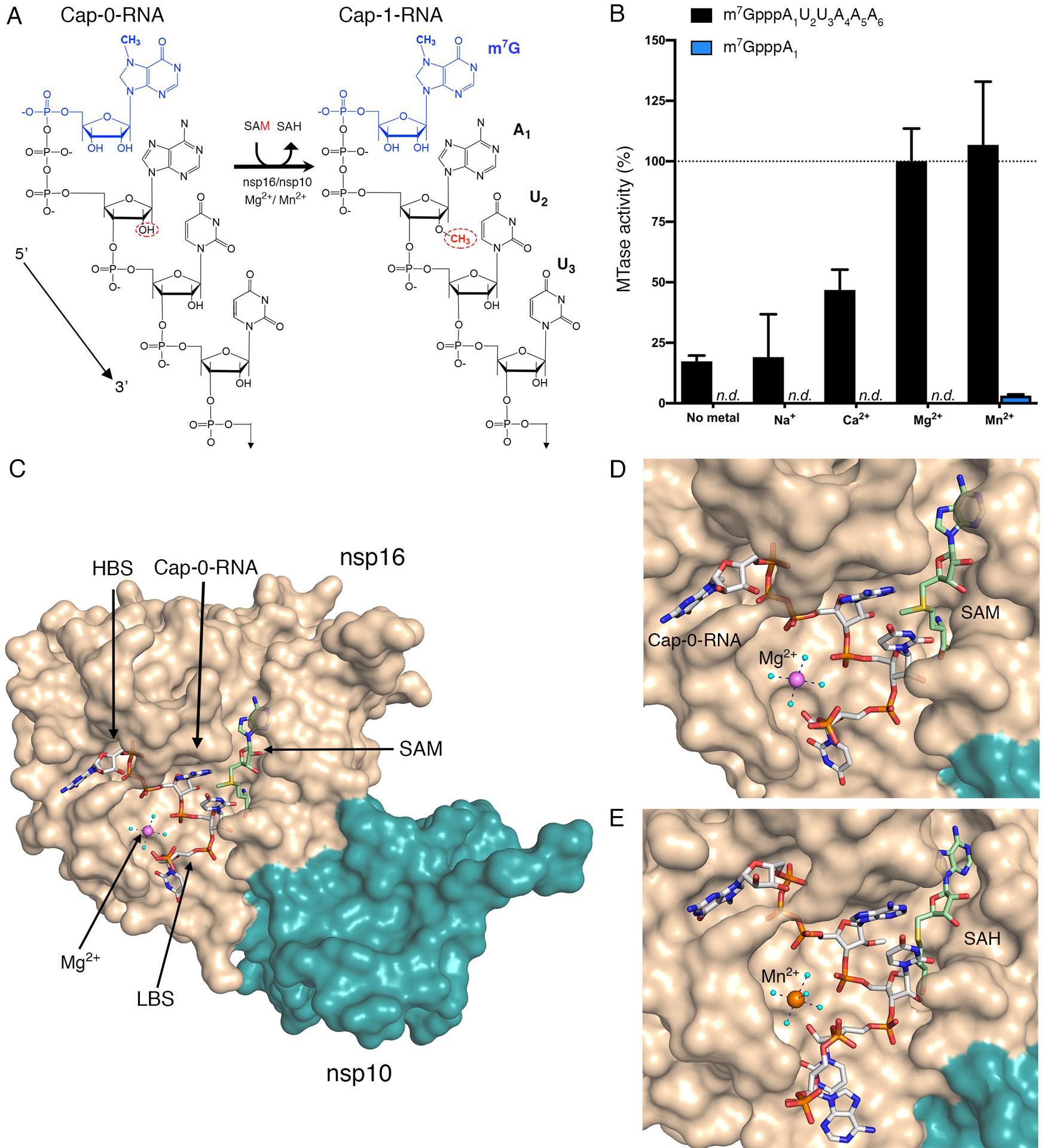
Metal ions catalyze 2′-*O*-methyl transfer and orient the Cap-0-RNA in the active site. **(A)** Schematic representation of the 2′-*O*-methyl transfer reaction generating Cap-1-RNA from Cap-0-RNA. The methylated guanosine cap (m^7^G) moiety is colored in blue, and the methyl group added to the A_1_ 2′-OH position from the methyl donor SAM is in red. The remainder of the RNA structure with the first three labeled ribonucleotides is in black. **(B)** The MTase-Glo luminescence assay results for Cap-0-RNA (m^7^GpppAUUAAA) and Cap-0 analog (m^7^GpppA) as substrates. *n.d*., no activity detected. The nsp16-nsp10 activity with Cap-0-RNA, SAM, and Mg^2+^ was selected as a reference (100%), and all measured activities were normalized to this value. All values are means ± the standard deviation for biological triplicates conducted in two separate experiments using two independent preparations of nsp16-nsp10 (*n*=6). (**C**) The overall view of nsp16-nsp10 in complex with Mg^2+^, Cap-0-RNA, and SAM, (PDB 7JYY). The high-affinity binding site (HBS) and the low-affinity RNA binding (LBS) are labelled. (**D and E**) Close-up views of nsp16-nsp10 in complex with (D) Mg^2+^, Cap-0-RNA and SAM (PDB 7JYY) or with (D) Mn^2+^, Cap-1-RNA, and SAH (PDB 7L6R). The nsp16 and nsp10 proteins are represented as solvent-exposed surfaces in tan and teal, respectively. Capped RNAs, SAM, and SAH are shown as sticks. Carbons are in grey for capped RNAs and in green for SAM and SAH; oxygens are in red, nitrogens are in blue; phosphates are in orange; sulfur is in yellow. Metal ions are shown as large spheres colored in purple for Mg^2+^ and orange for Mn^2+^. Water molecules are small, cyan spheres. Hydrogen bonds between metal ions and waters from the first hydration sphere are shown as black dashed lines.

Coronavirus genome replication and transcription processes are confined to viral-specific organelles formed from endoplasmic reticulum membranes (8, 9) during early stages infection, and probably mitochondrial, endosomal, and Golgi membranes during viral particle assembly (8–10). Thus, nascent coronavirus mRNAs do not have access to the host mRNA capping and methylation machinery in the nucleus and instead encode their own capping machinery. The viral replication helicase nsp13 also has 5′-RNA triphosphatase activity that removes the 5′-γ-phosphate from the nascent ppp-RNA (11). The RNA-dependent RNA-polymerase nsp12 can then transfer a guanosine monophosphate to the 5′-end of the mRNA (12, 13), and this guanosine is then methylated by the nsp14-nsp10 heterodimer to generate *N*^7^-methylated Cap-0-RNA (m^7^GpppA_1_-RNA). Finally, to form the Cap-1-RNA, a methyl group is transferred from *S*-adenosylmethionine (SAM) to the 2′-OH of the first adenosine ribonucleotide. For coronaviruses, this last reaction is catalyzed by the 2′-*O*-methyltransferase (MTase), a heterodimeric complex of nsp16 with the activator nsp10 (Fig. 1A) (11, 14, 15). The coronavirus 2′-*O*-MTase is a validated antiviral drug target, because the formation of the nsp16-nsp10 complex can be blocked by peptides and protect mice from lethal challenge with mouse hepatitis virus (MHV) (16, 17).

Biochemical studies of the RNA MTases from SARS-CoV and MERS-CoV demonstrated that methyl transfer is activated by divalent cations. SARS-CoV nsp14 is activated by Mn^2+^, but not by Mg^2+^ (11), whereas nsp16 can be activated by Mn^2+^ or Mg^2+^ and to a lesser extent by Ca^2+^ (11, 18–20). These data are consistent with known requirements for divalent cations by other viral RNA MTases, including dengue virus (DENV) NS5, which is stimulated by Mg^2+^ (21). Furthermore, studies of nsp16 from SARS-CoV and MERS-CoV have demonstrated that the methyl transfer reaction is most efficient in vitro with an RNA substrate comprised of at least five ribonucleotides (11, 19, 20, 22). However, the role of metals in nsp16 catalysis and the influence of mRNA length on the reaction is not clear, particularly because no structures have been solved for any coronavirus nsp16 enzyme with bound metals or RNA. To support drug discovery efforts targeting nsp16, researchers around the globe have determined crystal structures of SARS-CoV-2 nsp16-nsp10 in complex with variety of ligands (23–29), complementing prior structural biology observations for this enzyme from SARS-CoV and MERS-CoV (19, 20, 30). This information has facilitated detailed examination of the SARS-CoV-2 2′-*O*-MTase and revealed conformational flexibility of loop 1 and loop8-η3-loop9, loops that border the Cap-0 binding site (25, 26). Despite this extensive structural information, a major gap exists in understanding of the role of metal ions in the nsp16 2′-*O*-methyl transfer reaction and the position of ribonucleotides in the RNA binding groove because structures of the enzyme in complex with RNA and metals have not been reported. Herein, we present structures of the SARS-CoV-2 2′-*O*-MTase nsp16-nsp10 heterodimer in complex with substrates and products in the presence of divalent cations. Our structures reveal important information regarding interactions of nsp16 residues with the capped RNA, the influence of a coronavirus MTase-specific four-residue insert on the conformation of capped RNA, and the role of metal ions in 2′-*O*-methyl transfer.

## RESULTS

### SARS-CoV-2 nsp16 activity requires extended capped RNA and divalent cations.

We launched this research by confirming that the SARS-CoV-2 2′-*O*-MTase activity requires divalent cations. We used a custom-synthesized Cap-0-RNA substrate comprised of the *N*^7^-methylated guanosine (m^7^G) attached through a triphosphate bridge to a short RNA (AUUAAA), which matches the naturally occurring ribonucleotides at the 5′-end of SARS-CoV-2 mRNAs (31). At a concentration of 2 mM, both Mg^2+^ and Mn^2+^ increased MTase activity on this substrate (Fig. 1B). In contrast, 2 mM Ca^2+^ yielded only 50% of the activity observed with Mg^2+^ and Na^+^ did not stimulate activity at 3 mM (Fig. 1B). These data are consistent with observations for SARS-CoV nsp16 (11, 19, 20). The activity of SARS-CoV-2 nsp16 with the Cap-0-RNA substrate (m^7^GpppAUUAAA) was over 10-times higher than that with the Cap-0 analog (m^7^GpppA) (Fig. 1B).

Isothermal calorimetry (ITC) measurements showed that, although it is a poor substrate for catalysis, m^7^GpppA does bind to nsp16-nsp10 (K_d_ = 6.6 ± 0.3 μM) with 3-fold higher affinity than to nsp16 alone (K_d_ = 28.0 ± 5.5μM) (fig. S1, C and E; table S1). In contrast, m^7^GpppG bound only to the nsp16-nsp10 heterodimer but not to nsp16 alone (K_d_ = 20.0 ± 2.7 μM) (fig. S1 D; table S1). We also determined the binding affinities for the methyl donor SAM and the product *S-*adenosylhomocysteine (SAH) for nsp16 and the nsp16-nsp10 heterodimer (fig. S1, A and B; table S1). Neither SAM nor SAH bound to nsp16 alone, but both SAM and SAH bound to the nsp16-nsp10 heterodimer with K_d_ values of 6.9 ± 1.3 μM and 13.0 ± 1.2 μM, respectively. These biochemical and ITC studies together, as well as work by others (11, 24, 26, 32), indicate that the Cap-0 analog is capable of binding to nsp16 alone and that nsp10 greatly enhances the binding affinity. However, a capped short RNA and the presence of Mg^2+^ or Mn^2+^ stimulates the rates of catalysis.

### Crystal structures of the nsp16-nsp10 heterodimer with capped RNA and metal ions.

To gain insight into how metal ions stimulate catalysis, we took a structural biology approach. Crystals of nsp16-nsp10 in complex with SAM from different crystallization conditions were soaked with the custom-synthesized m^7^GpppAUUAAA substrate in the presence of Mg^2+^ or Mn^2+^. Multiple datasets were collected and, ultimately the three with the highest resolution and the best data statistics were selected for further analysis (table S2). Crystal #1 (PDB 7JYY) was soaked with substrates in the presence of 5 mM MgCl_2_ for 1.5 hours. In this crystal we observed Cap-0-RNA, SAM, and Mg^2+^ (Fig. 1C, D). Crystal #2 (PDB 7L6R) was soaked with substrates in the presence of 20 mM MnCl_2_ for 6 hours. In this crystal we observed Mn^2+^ and products of the reaction, Cap-1-RNA and SAH, indicating that the methyl transfer occurred in the crystal (Fig. 1E). Crystal #3 (PDB 7L6T) was soaked with substrates in the presence of 500 mM MgCl_2_ for 6 hours. In this crystal we observed two Mg^2+^ ions and the products Cap-1-RNA and SAH. The first Mg^2+^ occupied the same metal binding site as in Crystals #1 and #2, and the second Mg^2+^ directly interacted with phosphate groups of the capped RNA (fig. S2). The number of ribonucleotides in Crystal #1 and #3 included the m^7^G cap, first three ribonucleotides, and the phosphate group of A_4_. Crystal #2 contained the m^7^G cap, the first four ribonucleotides, and the phosphate group of A_5_.

### Substrates are properly aligned when the capped short RNA is present.

**Fig. 2.**
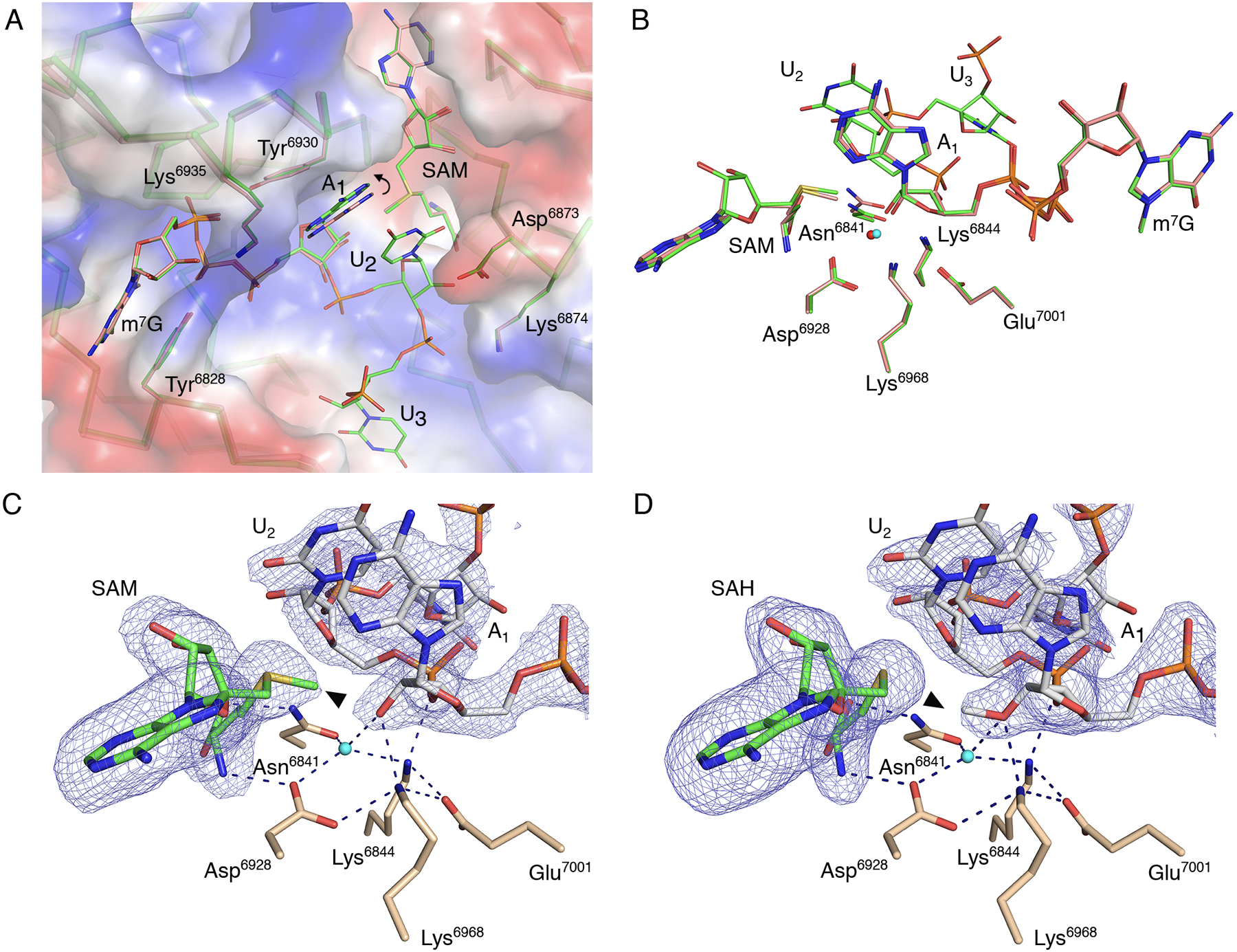
The catalytic site of the SARS-CoV-2 2′-*O*-MTase. (**A**) Superimposition of nsp16 with the Cap-0 analog (PDB 6WVN, pink) and nsp16 with the Cap-0-RNA (PDB 7JYY, green) structures with plotted electrostatic surface shown in blue (positive charge) and red (negative charge). Selected residues of nsp16, SAM, the Cap-0 analog, and Cap-0-RNA are labeled and shown as sticks. Carbons are in salmon and green for Cap-0 analog and Cap-0-RNA, respectively, with oxygens in red, nitrogens in blue, and sulfurs in yellow. Repositioning of the A_1_ base is marked with a curved arrow. (**B**) Catalytic residues of nsp16, Cap-0 analog, Cap-0-RNA, and conserved water for the same structures and same color scheme as in (**A**), with waters shown as small spheres in red and cyan for structures 6WVN and 7JYY, respectively. (**C and D**). Wall-eyed pseudo-stereo view of the active sites for complexes of (C) nsp16-nsp10 with Cap-0-RNA and SAM (PDB 7JYY) and (D) nsp16-nsp10 with Cap-1-RNA/SAH (PDB 7L6R). The catalytic site residues, SAM, and capped RNAs are labeled and shown as stick models with atoms colored in beige, green, and grey for carbons of nsp16, SAM, and capped RNA, respectively, with red for oxygens, blue for nitrogens, yellow for sulfurs. Conserved catalytic waters are shown as cyan spheres, hydrogen bond interactions as black, dashed lines, and the omit |Fo-Fc| electron density maps contoured at the 3σ level as blue mesh. The methyl group of SAM and Cap-1-RNA are marked with black triangles.

An overlay of the previously reported structures of SARS-CoV-2 nsp16-nsp10 with bound Cap-0 analog (PDB 6WRZ (25)) and nsp16-nsp10 with Cap-0-RNA (PDB 7JYY, this study) revealed that they are very similar with a root-mean-square-deviation of 0.33 Å (Fig. 2A). We previously showed that the cap binding site, also called the high-affinity binding site (HBS), is bordered by flexible loops that adopt an open conformation upon Cap-0 analog binding (25). Interactions of the nsp16 residues with the Cap-0 analog and Cap-0-RNA are similar in both structures. The m^7^GpppA in the HBS is stabilized by stacking of the m^7^G and A_1_ bases with Tyr^6828^ and Tyr^6930^ residues, respectively (Fig. 2A). The O2′ of the A_1_ ribose interacts with the conserved nsp16 catalytic residues (19, 20) as well as with a conserved water molecule we previously identified (25). The interactions between Asn^6841^, SAM, and O2′ from the A_1_ are also consistent between these structures (Fig. 2B).

Of particular note was the space between the Tyr^6930^ and Asp^6873^ side chains, which is occupied by the A_1_ base in the Cap-0 analog structure. In the Cap-0-RNA structure, this space accommodates the stacked bases of A_1_ and U_2_, with the U_2_ base forcing repositioning of the A_1_ ribonucleotide (Fig. 2A). Superposition of the Cap-0 and Cap-0-RNA structures revealed that this repositioning involves both a 0.6 Å shift of the A_1_ base towards the side chain of Tyr^6930^ without notable changes in the positions of Asn^6873^ and Tyr^6930^, as well as a 0.4 Å decrease in the distance between O2′ of A_1_ and the SAM methyl group, which occurs without apparent changes in the position of catalytic residues (Fig. 2B). The movement and alignment of the A_1_ O2′ atom toward the methyl group of SAM may explain why the additional ribonucleotides increase the efficiency of the methyl transfer reaction (33–35).

Although the superposition of Cap-0 and capped RNA structures revealed differences in the position of the first adenosine, no deviations were observed between Cap-0-RNA and Cap-1-RNA conformations (Fig. 2 A,B), indicating that the A_1_ base is not repositioned after the methyl transfer. The structures are essentially identical with the only difference being the methyl group, which moves from SAM to the A_1_ ribose hydroxyl group during methyl transfer (Fig. 2 C,D).

### Interaction of capped RNA ribonucleotides with residues of the low-affinity binding site

**Fig. 3.**
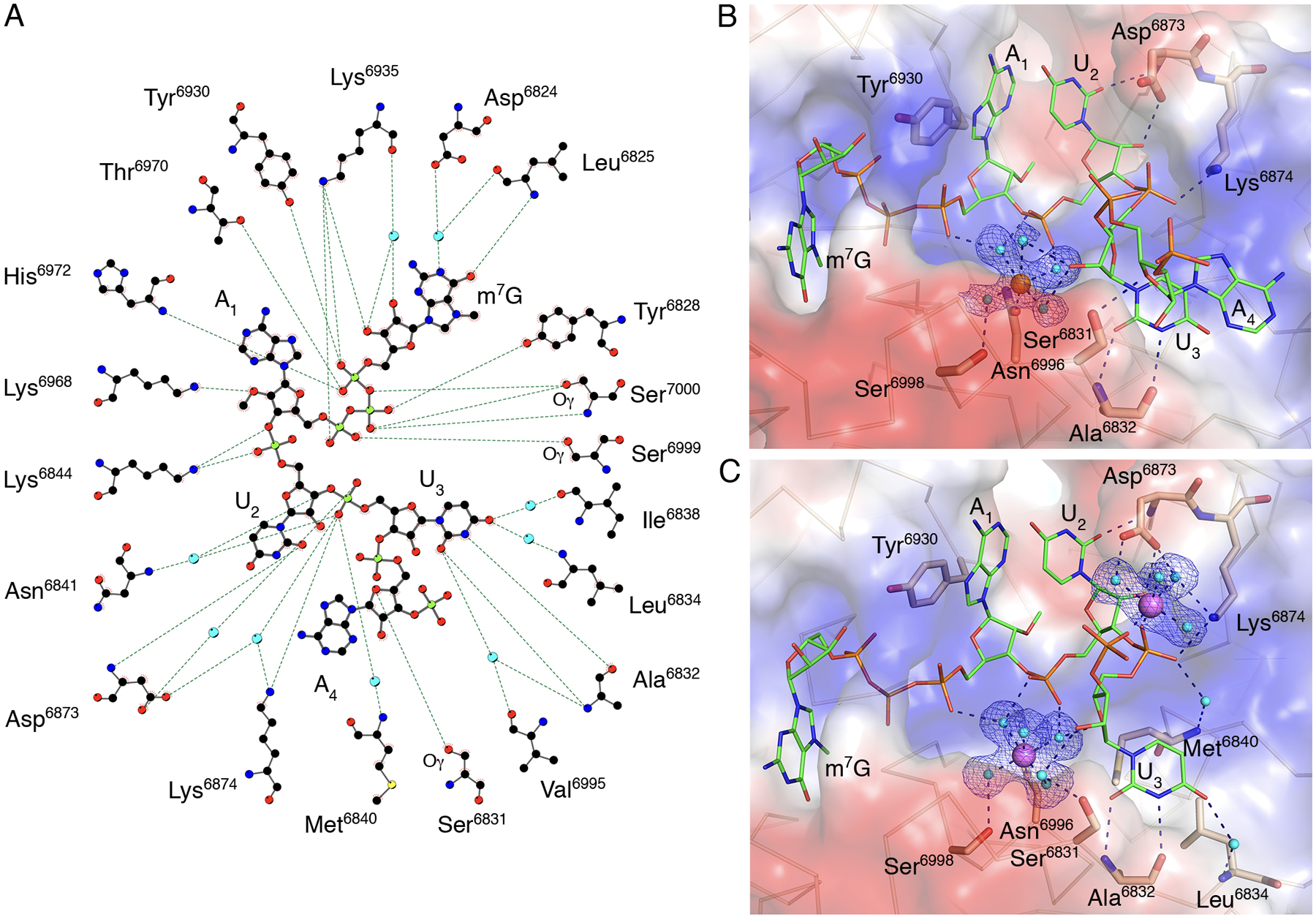
Close alignment of the 2′-OH and methyl group of SAM in presence of RNA. (**A**) A ball-and-stick representation of the hydrogen bonding network (dashed lines) between m^7^GpppA-RNA and nsp16 residues in Crystal #2. Carbons are shown in black, nitrogens in blue, oxygens in red, phosphates in light green, sulfurs in yellow, and waters in cyan. (**B and C**) A detailed view of the hydration sphere of (B) Mn^2+^ (orange sphere) in Crystal #2 and (C) Mg^2+^ (purple spheres) in Crystal #3, mapped on the electrostatic surface of nsp16 (blue and red) and their interactions with water (cyan spheres) and nsp16 residues (beige sticks). The Cap-1-RNA is represented as sticks, with carbons in green, oxygens in red, nitrogens in blue, and phosphates in orange. Hydrogen bond interactions are shown as black dashed lines and the omit electron density maps as blue mesh.

The structures in complex with capped RNAs also revealed the importance of the low-affinity binding site (LBS) residues for the conformation of the mRNA in the catalytic site. The best resolved and most complete electron density for capped RNA was observed in Crystal #2. The position of U_2_ in the active site is “locked” by multiple interactions (Fig. 3A). The phosphate group of U_2_ interacts with water molecules from the hydration sphere of the metal ion and the side chain nitrogen of Lys^6844^; the O2 atom of the U_2_ base interacts with the main chain nitrogen of Asp^6873^, and the O2′ of the U_2_ ribose makes direct interactions with one of the oxygens of the side chain of Asp^6873^ (Fig. 3B). The phosphate group of U_3_ interacts directly with the side chain nitrogen of Lys^6874^ and forms water-mediated interactions with residues Asp^6873^, Lys^6874^, Met^6840^, and Asn^6841^. The base of U_3_ interacts directly with the main chain oxygen and nitrogen atoms of Ala^6832^ and forms a water bridge interaction with the nitrogen of the main chain of Leu^6834^. The whole nucleotide A_4_ and phosphate group of A_5_, the last ordered part of the capped RNA in the Crystal #2 structure, are solvent-exposed and connected to the protein by a hydrogen-bond interaction between O4′ of the A_4_ and the side chain oxygen of Ser^6831^ (Fig. 3B). The stacking interactions between bases of U_3_ and A_4_ define the position of the A_4_ nucleotide. It is unknown if the conformation of A_4_ reflects the natural interaction of nucleotides, or if a longer mRNA would form different contacts with the nsp16-nsp10 heterodimer. However, the position of m^7^G and first three nucleotides of the capped RNA closely match in all three structures and likely represent the accurate binding mode for this part of the capped RNA.

### Metal ions stabilize the capped RNA in the nsp16 active site

The primary metal binding site is located near the HBS (Fig. 1C), with either Mg^2+^ or Mn^2+^ occupying the same site with similar interactions (Fig. 3B,C). The metal ions make both direct and water-mediated interactions with side chains of nsp16 residues and the backbone of the capped RNA. The best electron density maps were observed for Crystal #3 with two magnesium ions, both of which have near-ideal octahedral geometry (Fig. 3C). The Mg^2+^ in the primary metal binding site is coordinated in part by interactions with phosphate groups of the triphosphate bridge linking the cap to A_1_, the phosphate group of U_2_, and the ribose of U_3_. The second Mg^2+^ directly interacts with the U_3_ and A_4_ phosphate group oxygens and through water molecules with the side chain oxygens of the Asp^6873^ and the side chain nitrogen of Lys^6874^ (Fig. 3C). Thus, the metal ion that occupies the primary metal binding site of nsp16 properly aligns capped RNA with SAM for an efficient methyl transfer reaction. The role of metal ions in facilitating orbital alignments for efficient catalysis has been demonstrated structurally for hydride transfer reactions (34). Structural evidence for the requisite orbital alignment of substrates in RNA 2’-*O*-MTases (33) and ribozymes (35) has also been observed.

### Asp^6873^ and Lys^6874^ alter the backbone conformation of the capped RNA in coronaviruses

**Fig. 4.**
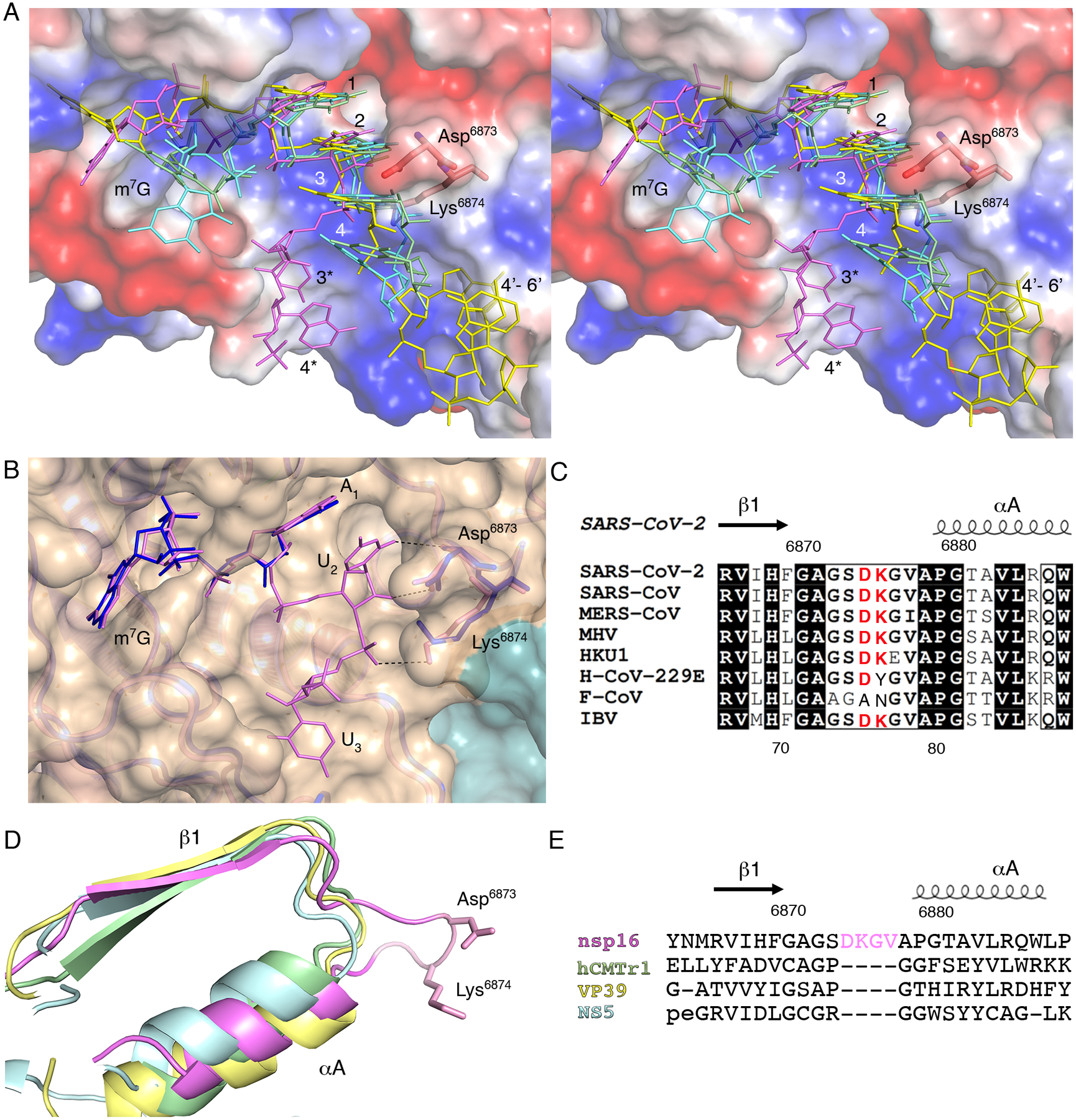
A four-residue loop in coronavirus nsp16 alters the orientation of the RNA substrate. (**A**) Wall-eyed stereo view of the superimposed capped RNAs mapped on the electrostatic potential surface of SARS-CoV-2 2′-*O*-MTase. RNAs are shown as sticks in bright pink (SARS-CoV-2), cyan (DENV NS5), yellow (VACV VP39) and green (hCMTr1). The nucleotides are labeled with numbers starting from m^7^G (position 0); 3* and 4* correspond to U_3_ and A_4_ in the Cap-1-RNA from SARS-CoV-2, 4’-6’ to A_4_-A_5_-A_6_ in the capped mRNA from VACV. (**B**) Superimposition of the RNA binding sites of nsp16-nsp10 with Cap-0-RNA (SARS-CoV-2, magenta) and Cap-0 analog (MERS-CoV, blue). Protein chains are shown as ribbons in pink for SARS-CoV-2 (PDB 7JYY) and blue for MERS-CoV (PDB 5YNM) overlayed on the semitransparent solvent-exposed surface of nsp16 (beige) and nsp10 (teal). Direct hydrogen bond interactions of Asp^6873^ and Lys^6874^ with U_2_ are shown as black, dashed lines. (**C**) Multiple sequence alignment of the loop region between β1 and αA from different coronaviruses, with Asp^6873^ and Lys^6874^ highlighted in red. SARS-CoV, severe acute respiratory syndrome; MERS-CoV, Middle East respiratory syndrome; MHV, murine hepatitis virus (MHV); HKU1, human coronavirus 1; H-CoV-229E, human coronavirus 229E; F-CoV, feline coronavirus; IBV, infectious bronchitis virus (IBV). (**D and E**) Structural and sequence alignments of nsp16 in pink, hCMTr1 in green, VP39 in yellow, and NS5 in cyan. Protein chains are shown as cartoon models with conserved Asp^6873^ and Lys^6874^ from nsp16 shown as sticks and the insertion loop highlighted in pink.

Comparison of the RNA-binding site and the capped RNA conformation from the SARS-CoV-2 nsp16-nsp10 structure (PDB 7L6R) and the crystal structures of 2′-*O*-MTases with capped RNA for DENV NS5 (PDB 5DTO (21)), vaccinia virus (VACV) VP39 (PDB 1AV6 (36)), and human cap methyltransferase hCMTr1 (PDB 4N48 (37)) revealed that the conformation of the m^7^G and the location of the cap-binding pockets relative to the active sites are dramatically different (fig. S3A–D). In the nsp16-nsp10 and VP39 structures, the *N*^7^-methyl groups are nestled in the HBS pocket (fig. S3 A,C). In contrast, in NS5 and hCMTr1, for which methylation at the *N*^7^ position of the G_0_ is not required for the 2′-*O*-MTase activity (37), the *N*^7^-methyl groups are pointed toward the solvent (fig. S3 B,D). Although the m^7^G positions do not overlap, nucleotides N_1_ and N_2_ in all these structures are closely matched, which is consistent with the conserved mechanism of action and the structure of the catalytic site (Fig. 4A). In all but the nsp16-nsp10 structure, the N_2_ nucleotide is sandwiched between the N_1_ and N_3_ by base stacking interactions and these three nucleotides have limited interactions with the 2′-*O*-MTase residues of RNA binding grove. In the nsp16-nsp10 structure all functional groups of the nucleotides N_2_ and N_3_ are involved in an integrated and complex network of hydrogen bond interactions with residues of the LBS. The U_2_ base and the ribose directly interact with main chain and side chain atoms of Asp^6873^. The Asp^6873^ side chain occupies the space that is filled by N_3_ in all other structures and forces the U_3_ nucleotide to move to the opposite side of the RNA binding groove. The base of U_3_ is involved in direct and water-mediated interactions with Ala^6832^ and Leu^6834^ while the phosphate group interacts with Lys^6874^ (Fig. 3 B,C). The superposition of SARS-CoV-2 nsp16-nsp10 (PDB 7JYY) with MERS-CoV (PDB 5YNM (38)) revealed almost identical conformations of the proteins and the promontory Asp^6873^ and Lys^6873^ residues, as well as the position of the Cap moiety (Fig. 4B). Alignment of the amino acid sequences of nsp16 from representative coronaviruses (Fig. 4C; fig. S4, A and B) showed that Asp^6873^ is conserved across the coronaviruses, except for feline coronavirus (F-CoV). Structural alignment of nsp16 with NS5, VP39, and hCMTr1 revealed that two important residues, Asp^6873^ and Lys^6874^, are located in a four-residue loop that connects β1 and αA (Fig. 4D). This four-residue insert is uniquely present in all coronavirus nsp16 MTases and is absent from other MTases (Fig. 4E).

**Fig. 5.**
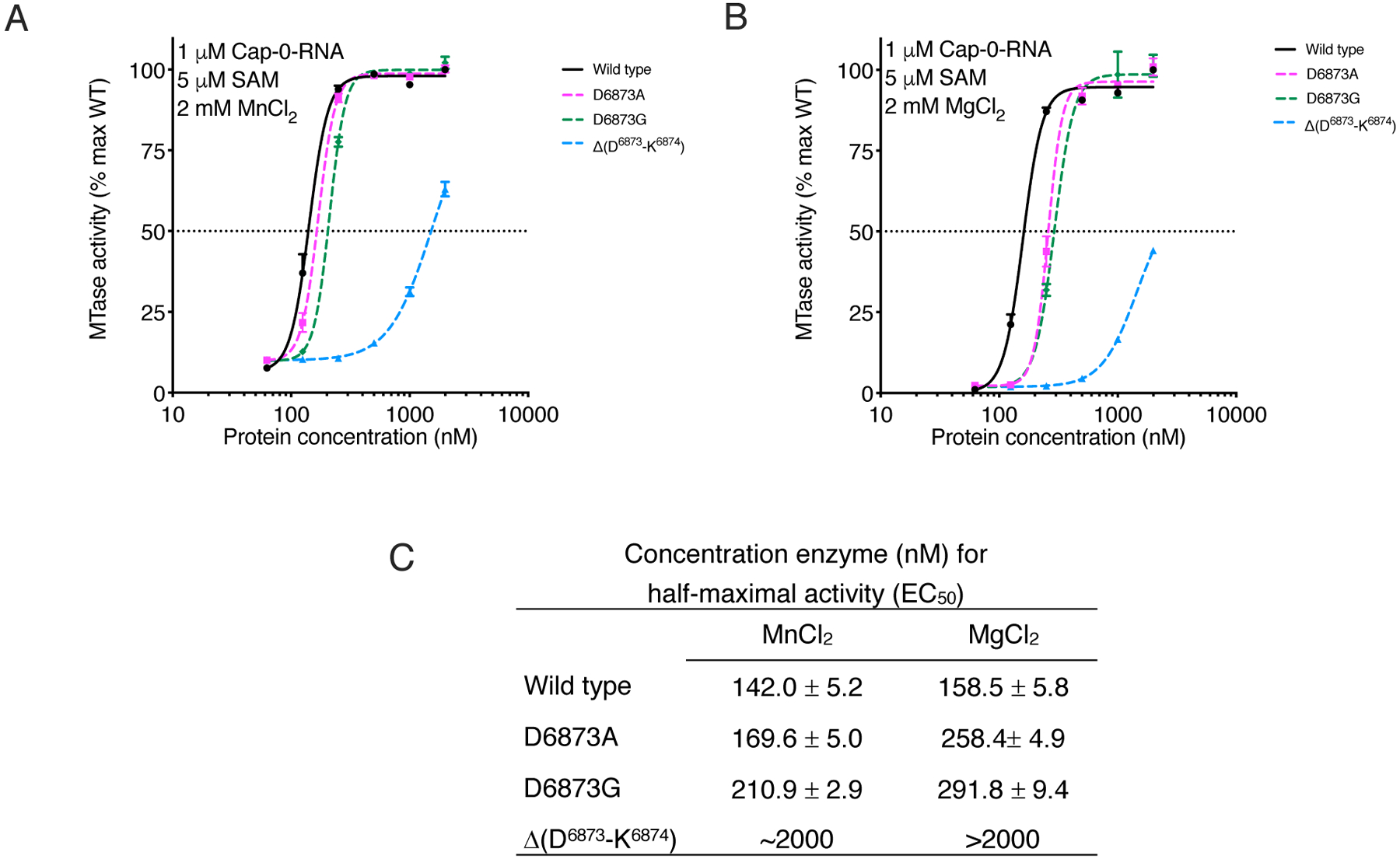
Mutations at Asp^6873^ and Lys^6874^ reduce the efficiency of the nsp16 methyltransfer reaction. **(A and B)** Plot of nsp16 methyl transfer activity across a concentration range for the indicated wild-type (WT) and mutant nsp16-nsp10 heterodimer complexes, plotted as the percentage of maximum (max) activity of the WT enzyme. Data are mean and standard deviation for biological triplicates conducted in two separate experiments. (*n*=6). Assays were conducted in presence of MnCl_2_ (A) or MgCl_2_ (B). Black horizontal dashed line indicates 50% activity. (**C**) Calculated enzyme concentration for half-maximal activity (EC_50_).

To test the effect of this insert on nsp16 MTase activity, we prepared nsp16 proteins with D6873A or D6872G amino acid substitutions or with a deletion of Asp^6873^ and Lys^6874^ within the loop (Δ(D^6873^-K^6874^)). Overall, Mn^2+^ stimulated the methyl transferase activity of nsp16 better than did Mg^2+^ for both the wild-type and mutant nsp16 proteins (Fig. 5, A and B). At a 30 nM protein concentration in the presence of Mn^2+^, we observed 10% of the maximum activity for all proteins, whereas only 2% activity was observed in the presence of Mg^2+^. A similar pattern was detected when we measured values for maximum activity, which was reached at an enzyme concentration of 500 nM in the presence of Mn^2+^, but required 1 mM enzyme in the presence of Mg^2+^ (Fig. 5A,B). The EC_50_ values for activity of the D6873A and D6873G mutants in the presence of Mn^2+^ were shifted higher than values obtained for the wild-type enzyme (Fig. 5C). These shifts in EC_50_ values were more evident in the presence of Mg^2+^. Notably, the Δ(D^6873^-K^6874^) mutant showed a much lower amount of activity compared to the D6873A and D6873G mutants and the wild-type enzyme. In fact, at enzyme concentrations as high as 1 μM , the activity of Δ(D^6873^-K^6874^) reached only 45% and 65% of that of the wild-type max activity in the presence of Mn^2+^ or Mg^2+^, respectively. The results of activity assays support our structural finding regarding the importance of the four-residue loop that is unique for coronavirus nsp16 MTase as well as a preference for stimulation of the methyl transfer reaction by Mn^2+^.

## DISCUSSION

In eukaryotes, 5′ capping and subsequent 2′-*O*-methylation events are essential for RNA metabolism and protection from cell innate immunity responses (39). Coronaviruses and other viruses have evolved entire RNA capping and methylation machineries to modify their own RNAs, thereby increasing the translation of viral proteins and promoting the evasion of host immune responses (7, 40, 41). For this reason, the viral 2′-*O*-MTases are suitable targets for drug development. Thus far, all proposed *in silico* drugs that could target the residues of the LBS (19, 24, 26, 32) have relied on the crystal structures of DENV NS5, VACV VP39, and hCMTr1, indicating a knowledge gap for drug design.

The metal ion requirement for DENV, SARS-CoV, and MERS-CoV MTase activity is well known (11, 19, 20). However, the binding site of the metal ion and capped RNA has been shown only for the NS5 structure (21). In the NS5 structure, a Mg^2+^ ion directly interacts with all three phosphate groups that link the m^7^G with the A_1_, and thus neutralizes the negative charge of the triphosphate group. Three additional interactions through its hydration sphere with the bases of the capped RNA help maintain stacking interactions and stabilize the single-stranded RNA helix in the A-form. The described Mg^2+^ is not involved in any direct hydrogen bond interactions with the residues of the 2′-*O-*MTase domain. In all SARS-CoV-2 nsp16 structures reported here, the metal ions that occupy the primary metal binding site directly interact with Asn^6996^. This direct interaction “pins” the metal ion to the negatively charged “nest”. By neutralizing the negative charge, metal ions stabilize the unique conformation of the capped RNA and by direct interaction with Asn^6996^ help to hold the RNA in the RNA-binding site for methyl transfer.

Metal ions tested in the activity assays had different effects on the methyl transfer efficiency of SARS-CoV-2 nsp16. Na^+^ has a lower charge and Ca^2+^ longer metal-ligand distances than Mg^2+^ or Mn^2+^, which might yield a more extended capped RNA that is weakly attached to the binding site. We suspect this is why Na^+^ and Ca^2+^ poorly stimulated the methyl transfer reaction in our assays. Mg^2+^ had been considered as the primary catalytic co-factor and is reported as an important additive for nsp16 activity assays (11, 19, 20, 22). The results of our biochemical assays, by contrast, showed that Mn^2+^ had the highest stimulation effect on the methyl transfer reaction, and maximum activity was reached at lower concentrations of enzyme. It was also reported that Mg^2+^ ions in micro- to millimolar concentrations can promote the cleavage of nucleic acids (42), suggesting that Mn^2+^ is preferable compared to Mg^2+^. In addition, Mn^2+^ has a higher reported concentration than Mg^2+^ in the endoplasmic reticulum and Golgi, where the SARS-CoV-2 replication vesicles are formed (43, 44). Taken together, these findings suggest that Mn^2+^ is the natural co-factor for the methyl transfer reaction in SARS-CoV-2 and other coronaviruses. This information could be very helpful in design of specific inhibitors to target the metal binding site and impair the activity of the complex.

Comparison of the RNA-binding sites and the capped RNA conformations from the prior MTase structures of NS5, VP39, and HCMTr1 with those of nsp16-nsp10 complexes revealed major differences between them. These four structures can be grouped into two classes: the *N*^*7*^ of the m^7^G buried in the binding pocket, as observed in nsp16 and VP39, and *N*^7^ exposed to the solvent, as observed in NS5 and hCMTr1. Despite these differences, the superposition of these structures revealed a close match for the positions and the conformations of the first and the second nucleotides of capped RNA. This observation is in line with the conserved mechanism of the 2′-*O*-MTase reaction. The major differences in the conformations of the RNA are located in the extended part of the capped RNA where it interacts with the residues of the LBS. In NS5, VP39, and hCMTr1, the N_1_-N_3_ nucleotides of the capped RNA are stabilized by stacking interactions. In contrast, Asp^6873^ in nsp16 alters the position of the N_3_ and prevents the stacking of N_3_ to N_1_-N_2_, and Lys^6874^ stabilizes the position of the U_3_ phosphate. All functional groups of the nucleotides N_2_ and N_3_ are involved in an integrated and complex network of hydrogen bond interactions with residues of the LBS. These distinct differences were found to impact catalysis with mutation of Asp^6873^, leading to a reduced EC_50_ and deletion of Asp^6873^ and Lys^6874^ from the loop shifting the EC_50_ to the micromolar range.

A key advance of this work was the use of a custom-synthesized substrate RNA with ribonucleotides matched specifically to the sequences of the SARS-CoV-2 mRNA 5’-ends compared to earlier studies that employed poly(C) RNA (11, 19). Use of this substrate, however, introduced a limitation of the studies due to precious amounts of the compound, which prevented maximizing the substrate concentration for kinetic and binding assays. However, using fixed conditions, we were able to reproducibly determine the crystal structure of nsp16-nsp10 in complex with Cap-0-RNA and the product of the reaction, Cap-1-RNA. This substrate could be used to conduct biochemical assays at a fixed concentration of substrates to show the preference of the enzyme for Mn^2+^ over Mg^2+^, a requirement for the extended RNA, and the importance of the unique promontory loop in nsp16 for catalysis. These studies in total indicate that redirection of the RNA, which is also coordinated by metals, leads to close alignment of the methyl group for transfer with the target 2’-OH on the first adenosine, and this is a unique feature that is shared across coronaviruses. The structures reveal that the presence of a unique four-residue insert (D^6873^-K^6874^-G^6875^-V^6876^) that is conserved in all coronaviruses is critical for catalysis. The absence of this insert in mammalian methyltransferases makes this region a promising site for the design of selective RNA-like coronavirus-specific inhibitors.

## MATERIALS AND METHODS

### Protein expression, purification, and crystallization

Recombinant nsp16 and nsp10 proteins were purified from *Escherichia coli* and crystallized as previously described (25). DNA sequences for expression of mutant nsp16 protein were also generated by synthetic DNA in the same vector (Twist Biosciences) and purified following the prior established protocol (25). To form the nsp16-nsp10 heterodimers, the pure proteins were mixed at a 1:1 molar ratio at ~2 mg/ml in buffer (10 mM Tris-HCl (pH 8.3), 500 mM NaCl, 1 mM Tris(2-carboxyethyl) phosphine (TCEP), 2 mM MgCl_2_, and 5% glycerol) and incubated for 1 h, then dialyzed in crystallization buffer (10 mM Tris-HCl (pH 7.5), 150 mM NaCl, 2 mM MgCl_2_, 1 mM TCEP and 5% glycerol) for 2 h. SAM was added to a final concentration of 2 mM. Freshly purified pre-formed nsp16-nsp10 heterodimer was concentrated to 4.0 – 5.5 mg/ml and immediately set up for crystallization as 2-μl crystallization drops (1 μl protein: 1 μl reservoir solution) in 96-well crystallization plates.

### MTase activity assays

Custom synthesized Cap-0-RNA (m^7^GpppAUUAAA) was obtained from Bio-Synthesis, Inc (Lewisville, TX). Cap-0 analog (m^7^GpppA) was obtained from New England Biolabs (catalog #S1405L). The MTase activity was measured using the MTase-Glo^™^ Methyltransferase bioluminescence assay (Promega) (45) according to the manufacturer’s protocol in a suitable solution for SARS-CoV-2 MTase: 20 mM Tris (pH 8.0), 1 mM EDTA, 100 nM nsp16-nsp10 heterodimer, 1 μM excess of nsp10, 5 μM SAM, 300 nM Cap-0-RNA or Cap-0 analog and 3 mM MgCl_2_, MnCl_2_, CaCl_2_ or 3 mM NaCl as indicated. The reactions were incubated for 1 h at 37°C and stopped with 0.5% trifluoroacetic acid. The detection solution from the kit was then added and the mixture further incubated for 30 min at room temperature, followed by the addition of the developing solution. Luminescence was measured using a TECAN Safire2 microplate reader in arbitrary units and normalized assigning 100% to the activity in presence of Mg^2+^ and Cap-0-RNA. The average and the standard deviation of three measurements in two independent experiments using different protein purifications (*n*=6) was plotted as a histogram using GraphPad Prism v9.

For determination of EC_50_, the assay was modified for a 384-well plate format at the Northwestern University High Throughput Analysis Laboratory. Each nsp16-nsp10 heterodimer complex was serially diluted in solution as detailed above using the Mosquito® robot. Reactions were initiated by addition of the substrates (5 μM SAM and 1 μM Cap-0-RNA) using the Mantis® liquid handler. After 1 h at 37°C, the reactions were coupled with the detection and developing solutions using Mantis® liquid handler and incubated as described above. Luminescence was measured in a TECAN Infinite M1000. The blank was defined as containing all reagents except Cap-0-RNA and this value was subtracted for each corresponding point. The values were then reported as the percentage of MTase activity compared to the wild type under the same conditions. Assays were conducted in triplicate and the experiment was independently repeated (*n*=6). EC_50_ and the standard error were calculated using four parameter logistic curve in GraphPad Prism v9 at 95% confidence interval.

### Isothermal Titration Calorimetry (ITC)

Binding affinity was determined using a MicroCal PEAQ-ITC system (Malvern, Worcestershire, UK) at 25 °C. The sample cell volume was 200 μL and the total syringe volume was 40 μL. For each titration, the first injection was performed using 0.4 μl which was then followed by 18 additional injections at 2 μl per injection. The first injection was considered a void and was automatically removed from data analysis. Each injection was spaced by 120 s after a 60 s initial delay. SARS-CoV-2 samples of nsp10, nsp16 and nsp16-nsp10 were individually loaded into the sample cell and then titrated with either SAH, SAM, m^7^GpppA Cap analog or the m^7^GpppG Cap analog (New England Biolabs catalog #S1411L). All samples were dialyzed overnight in ITC buffer (200 mM NaCl, 50 mM HEPES (pH 8.0), 0.1 mM ZnCl_2_, and 1 mM DTT). The concentrations of SARS-CoV-2 nsp10 or nsp16 used in experiments were 200 M and 40 μM, respectively, and the concentrations of all substrates used were 500 μM. For titration experiments of the SARS-CoV-2 nsp16-nsp10, the two proteins were mixed to the final concentrations 25 μM and 200 μM, respectively, and incubated for 30 mins at room temperature. A titration of SARS-CoV-2 nsp10 into nsp16 was performed at the 8:1 ratio to ensure no enthalpy was detected for the complex formation alone. The SARS-CoV-2 nsp16-nsp10 was titrated with substrates SAH and SAM at 375 μM and each Cap analog at 280 μM. Individual titration data were analyzed with MicroCal PEAQ-ITC Analysis Software using a single-site binding model and non-linear curve fitting. Each experiment was performed in triplicate and the resulting values and standard error in the fitted parameters for N, K_d_, ΔH, ΔTS, and ΔG were obtained and are summarized in table S2.

### Crystal soaking experiments

Diffraction quality crystals grown from different conditions were transferred into 10-μl drops containing 5 mM SAM, 0.2 mM Cap-0-RNA, and into 10-μl drops with 5 mM SAM, 0.2 mM Cap-0-RNA and 5 mM MgCl_2_ or 20 mM MnCl_2_ in respective reservoir solutions and soaked for a different time periods from 5 minutes to 17 hours. Crystals were cryoprotected with 4 M sodium formate or 25% sucrose in respective reservoir solution and flash frozen in liquid nitrogen for data collection.

Crystal #1 was grown from 0.1 M sodium citrate (pH 5.6), 1.0 M ammonium dihydrogen phosphate, soaked for 1.5 h with 0.2 mM Cap-0-RNA, 5 mM SAM, 5 mM MgCl_2_ and cryoprotected with 4 M sodium formate. Crystal #2 was grown from 0.1 M citric acid (pH 5.0), 0.8 M ammonium sulfate and was soaked for 6 hours with 0.2 mM Cap-0-RNA, 5 mM SAM and 20 mM manganese chloride. Crystal #3 was grown from 0.1 M HEPES (pH 7.5), 0.5 M magnesium formate and was soaked for 6 hours with 0.2 mM Cap-0-RNA, 5 mM SAM. Crystals #2 and #3 were cryoprotected with 25% sucrose in respective reservoir solutions.

### Data collection, processing, structure solution and refinement

More than 30 crystals were screened, and 32 data sets were collected at the beam lines 21ID-D and 21ID-F of the Life Sciences-Collaborative Access Team (LS-CAT) at the Advanced Photon Source (APS), Argonne National Laboratory. Images were indexed, integrated and scaled using HKL-3000 (46).

Data quality and structure refinement statistics are shown in table S2. All structures were determined by Molecular Replacement with Phaser (47) from the CCP4 Suite (48) using the crystal structure of the nsp16-nsp10 heterodimer from SARS-CoV-2 as a search model (PDB 6W4H). The initial solutions went through several rounds of refinement in REFMAC v. 5.8.0266 (49) and manual model corrections using Coot (7). The water molecules were generated using ARP/wARP (50) followed by additional rounds of refinement in REFMAC. All structures were carefully examined, and three data sets were selected for further structural studies. For all structures, the Cap-0-RNA, SAM and Mg^2+^ were fit into electron density maps and further refined. Inspection of anomalous and Fourier difference electron density maps revealed that, for Crystal #1 the nsp16-nsp10 heterodimer formed the complex with the Cap-0-RNA, SAM and Mg^2+^, for Crystal #2 the complex was formed with the Cap-1-RNA, SAH and Mn^2+,^ and for Crystal #3 the complex was formed with the Cap-1-RNA, SAH and two Mg^2+^. In Crystals #1 and #3, no additional electron density was detected beyond phosphate group of A_4_. In Crystal #2, the presence of well-defined electron density near phosphate group of A_4_ allowed unambiguously extend the RNA model by adding sugar and base for A_4_ and phosphate group of A_5_. All structures were further refined with the Translation–Libration–Screw (TLS) group corrections, which were created by the TLSMD server (51). The quality control of the models during refinement and for the final validation of the structures were done using MolProbity (52) (http://molprobity.biochem.duke.edu/). The capped RNA has a 5′-5′ triphosphate linkage between m^7^G and A_1_ with non-canonical RNA backbone geometry, which resulted in low RNA backbone scores in MolProbity reports. All structures were deposited to Validated SARS-CoV-2 related structural models of potential drug targets (https://covid19.bioreproducibility.org/) and to the Protein Data Bank (https://www.rcsb.org/) with the assigned PDB 7JYY (Crystal #1), 7L6R (Crystal #2) and 7L6T (Crystal #3). Flexible parts of the structure, alternative side chain conformations, and partial water molecules were fit into electron density maps at a lower σ level, which resulted in overall high RSRZ scores for all structures. All figures with models of the structures were created in PyMOL open source V 2.1 (53); the diagram showing protein and capped RNA interactions was created in LigPlot+ (54).

### Structural, sequence alignment and phylogenic analysis

The PDB coordinates of SARS-CoV-2 nsp16 and nsp10 were analyzed using the FATCAT (55), POSA (56) and DALI (57) servers to perform structural alignments with MERS-CoV, NS5, hCMTr1 and VP39 cap RNA MTases. Generated PDB files were downloaded from the servers and modeled in PyMOL open-source v2.1. The protein sequence of 2′-*O*-MTases were obtained from the National Center for Biotechnology Information database; feline coronavirus (F-CoV, AGT52079), murine hepatitis virus (MHV, YP_009915686.1), Human coronavirus (HKU1, YP_460023.1), Human coronavirus 229E (H-CoV-229E, AGT21344.1:6464-6763), Infectious bronchitis virus (IBV, NP_066134.1:6328-6629), dengue virus nonstructural protein NS5 (NS5, NP_739590.2), *Homo sapiens* (hCMTr1, BAA07893.3 KIAA0082) and vaccinia virus VP39 (VP39, NC_006998.1). The multiple sequence alignment was performed using Clustal-O (https://www.ebi.ac.uk/Tools/msa/clustalo/) and merged with the coordinates of the structure with PDB code 7JYY using ESPript 3.x (58). The phylogenic tree was created using MacVector and processed in iTol (https://itol.embl.de)

## Supplementary Material

SM file

## Data Availability

Structures reported are available from the RCSB Protein Data Bank (www.rcsb.org) and from covid19.bioreproducibility.org with accession numbers 7JYY, 7L6R, and 7L6T. Recombinant protein expression plasmids are available from www.beiresources.org under their standard MTA agreement. All other data needed to evaluate the conclusions in the paper are in the paper or the Supplementary Materials.
