# Supplementary material for: Mn2+ coordinates Cap-0-RNA to align substrates for efficient 2′-O-methyl transfer by SARS-CoV-2 nsp16
Source: Sci Signal. Author manuscript; Available in PMC 2021 Dec 29. (PMC8432954; doi:10.1126/scisignal.abh2071)
Supplement: SM file [file NIHMS1735346-supplement-SM_file.docx]

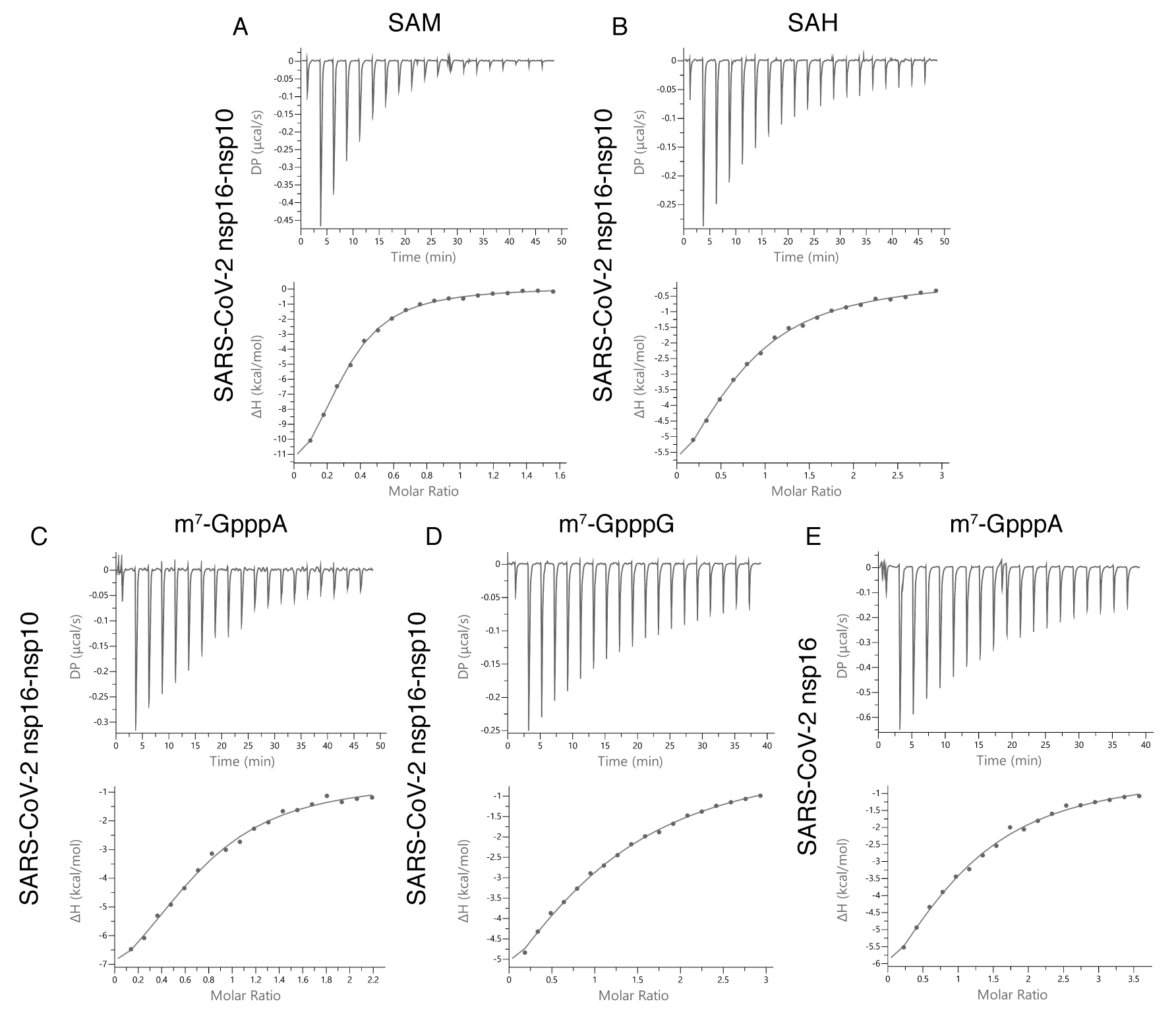


**Fig. S1. ITC titration curves for determination of K_d_ values for ligands interacting with the SARS-CoV-2 nsp16-nsp10 heterodimer or nsp16 alone**. The raw heat values (top) and the integrated heats of injection (bottom) are shown for the interaction of **(A)** SAM, **(B)** SAH, **(C)** m^7^-GpppA, and **(D)** m^7^-GpppG with the SARS-CoV-2 nsp16-nsp10 heterodimer and **(E)** the interaction of m^7^-GpppA with SARS-CoV-2 nsp16 alone. Data for each individual titration were fit to a single-site binding model, and a representative curve for each ligand is shown. Three independent titrations were ultimately performed for each ligand and the final fitted thermodynamic parameters were averaged and are provided in table S1 in the main text.

**
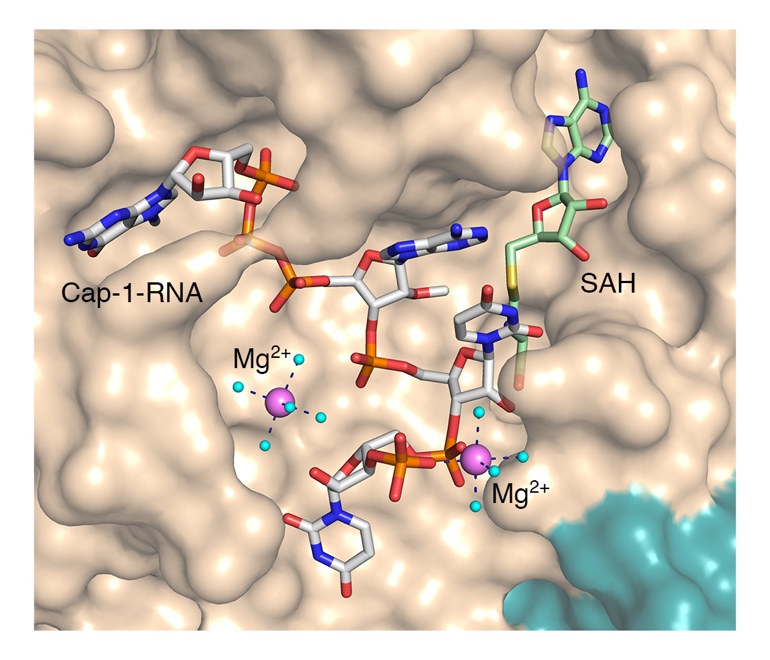
**

**Fig. S2. Close-up views of nsp16-nsp10 in complex with two Mg^2+^, Cap-1-RNA, and SAH.** The nsp16-nsp10 heterodimer (PDB code 7L6T) is represented as solvent-exposed surfaces in beige and teal, respectively. Cap-1-RNA and SAH are shown as sticks; carbons are in grey for capped RNAs, and green for SAH, oxygens in red, nitrogen in blue, phosphates in orange, sulfur in yellow. Mg^2+^ are shown as large spheres colored in purple. Waters are small spheres in cyan. Hydrogen bonds between metal ions and waters from the first hydration sphere are shown as black dashed lines.


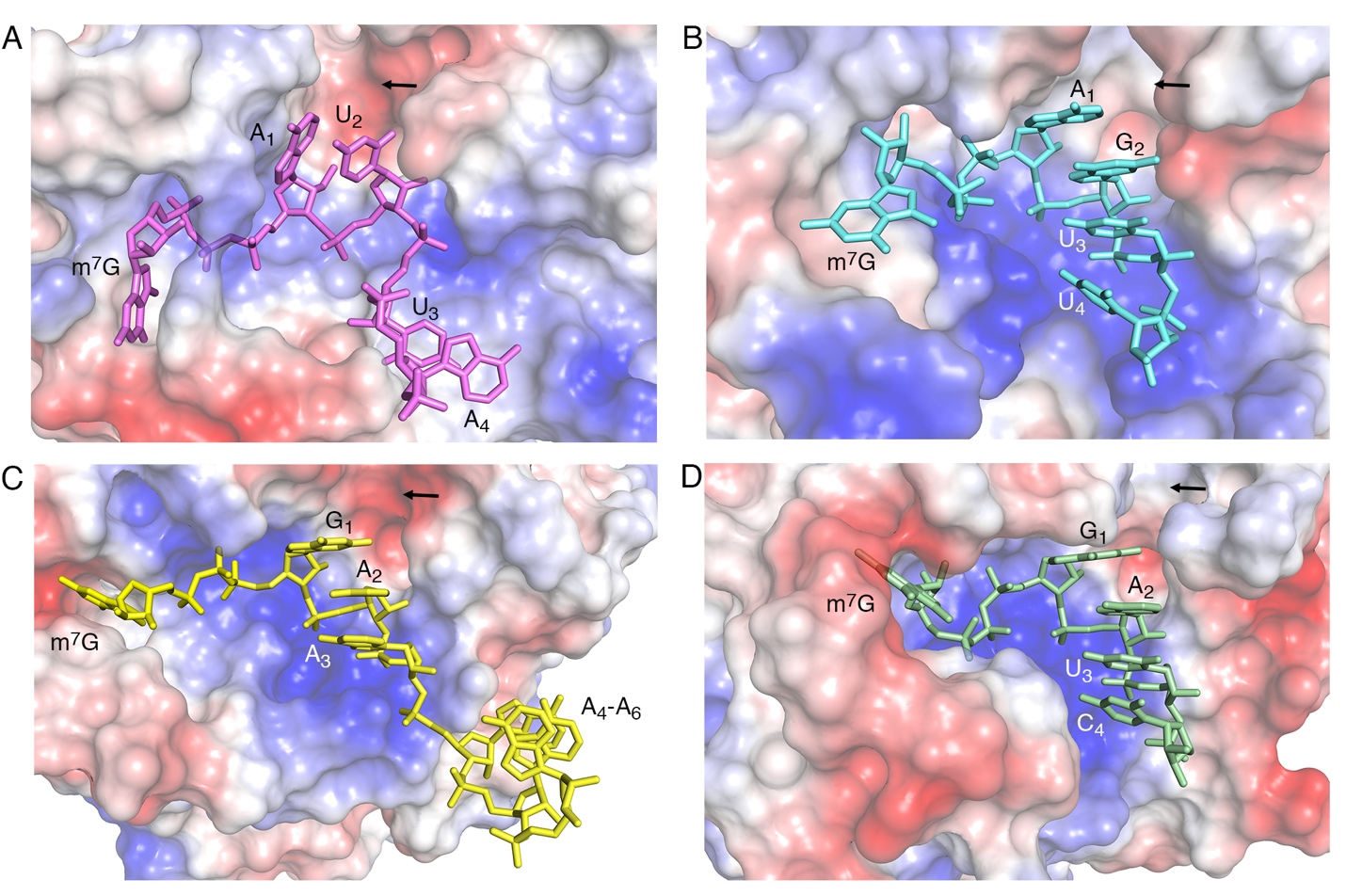


**Fig. S3.** **Comparison of the RNA-binding grooves of 2′-*O*-MTases.** Electrostatic potential surface representations of the 2′-*O*-MTases of **(A)** SARS-CoV-2 nsp16-nsp10 (PDB code 7L6R), **(B)** dengue virus NS5 (PDB code 5DTO), **(C)** vaccinia virus VP39 (PDB code 1AV6), and **(D)** the human MTase hCMTr1 (PDB code 4N48) with bound capped RNAs. Arrows indicate the SAM/SAH binding clefts and nucleotides are labeled as A for adenosine, G for guanosine, C for cytosine and U for uridine. The RNA bases are numbered starting from the 5′-guanine cap (m^7^G) (position 0). RNAs are shown as sticks in pink (SARS-CoV-2), cyan (NS5), yellow (VP39), and green (hCMTr1).

**
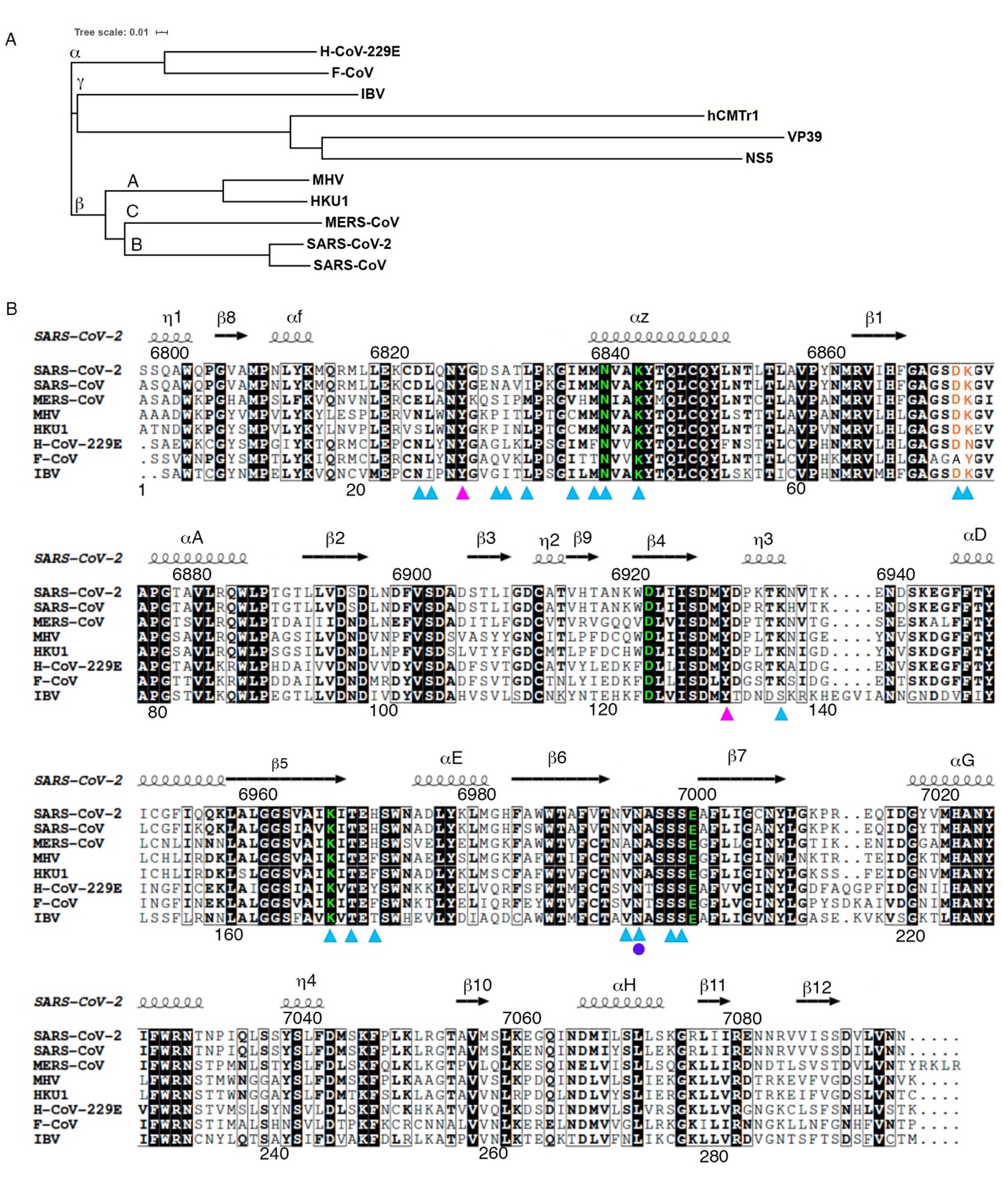
**

**Fig. S4.** **Conservation of 2′-*O*-Methyl transferases.** **(A)** Phylogenetic tree of MTases from coronaviruses, vaccinia virus VP3, dengue virus NS5, and human hCMTr1. **(B)** Primary amino acid sequence alignment of the 2′-*O*-MTases from different clades of coronaviruses showing the structural elements of nsp16 from SARS-CoV-2. Residues that are 100% conserved are shaded in black, catalytic residues are highlighted in green, residues that participate in Cap stacking are marked by pink triangles, RNA-protein interactions are marked by blue triangles, and the direct protein-metal interaction is indicated with purple circle. The conserved Asp^6873^ is colored orange. MHV, mouse hepatitis virus; H-CoV-229E, human coronavirus-229E; F-CoV, feline coronavirus; IBV, infectious bronchitis virus. The numbering of SARS-CoV-2 residues is on top and SARS-CoV is on the bottom.

**Table S1. ITC-derived thermodynamic parameters for the interactions of substrates with SARS-CoV-2 nsp10, nsp16, and the nsp16-nsp10 heterodimer.**

| Protein  (in ITC cell) | Substrate  (in syringe) | K_d_ (μM) | ΔH (kcal/mol) | TΔS  (kcal/mol) | ΔG  (kcal/mol) | N |
| --- | --- | --- | --- | --- | --- | --- |
| nsp10 | SAH | *n.d.** | | | | |
| nsp16 |  | *n.d.* | | | | |
| nsp16 + nsp10 |  | 13.0 ± 1.2 | -10.5 ± 0.5 | 3.6 ± 0.5 | -6.7 ± 0.1 | 0.64 ± 0.02 |
| nsp10 | SAM | *n.d.* | | | | |
| nsp16 |  | *n.d.* | | | | |
| nsp16 + nsp10 |  | 6.9 ± 1.3 | -17.0 ± 1.3 | 10.0 ± 1.5 | -7.0 ± 0.1 | 0.51 ± 0.01 |
| nsp10 | m^7^GpppA | *n.d.* | | | | |
| nsp16 |  | 28.0 ± 5.5 | -8.5 ± 1.4 | 2.2 ± 1.4 | -6.2 ± 0.1 | 1.2 ± 0.1 |
| nsp16 + nsp10 |  | 6.6 ± 0.3 | -8.7 ± 0.2 | 1.6 ± 0.2 | -7.10 ± 0.03 | 0.6 ± 0.1 |
| nsp10 | m^7^GpppG | *n.d.* | | | | |
| nsp16 |  | *n.d.* | | | | |
| nsp16 + nsp10 |  | 20.0 ± 2.7 | -10.0 ± 0.6 | 3.4 ± 0.7 | -6.5 ± 0.1 | 1.00 ± 0.03 |

^1^Values are derived from the average of three independent titrations. Standard error was calculated from the average of the three independent titrations. **n.d.=* enthalpy not detected.

Table S2. Data quality and structure refinement statistics.

| **Crystal** | **#1** | **#2** | **#3** |
| --- | --- | --- | --- |
| **PDB Accession Code** | **7JYY** | **7L6R** | **7L6T** |
| **Data Collection** |  |  |  |
| Wavelength (Å) | 0.97872 | 1.54980 | 1.12708 |
| Space group | *P3_2_21* | *P3_1_21* | *P3_1_21* |
| Unit cell parameters (Å; º) | *a=b=166.95, c=98.81;*  *α=β=90.00, γ=120.00* | *a=b=168.76, c=52.27;*  *α=β=90.00, γ=120.00* | *a=b=169.35, c=52.63;*  *α=β=90.00, γ=120.00* |
| Resolution range (Å) | 30.00-2.05 (2.09-2.05) | 30.00-1.98 (2.01-1.98) | 30.00-1.78 (1.81-1.78) |
| No. of reflections | 99,216 (4,938) | 59,455 (2,968) | 81,048 (3,493) |
| *R*_merge_ (%) | 8.3 (107.9) | 9.5 (120.3) | 9.0 (115.6) |
| Completeness (%) | 100.0 (99.8) | 100.0 (100.0) | 96.6 (84.2) |
| Rp.i.m.^a^ | 0.032 (0.415) | 0.032 0.411) | 0.036 (0.513) |
| CC_1/2_^b^ | 0.996 (0.765) | 0.997 (0.663) | 0.996 (0.560) |
| 〈*I*/*σ*(*I*)〉 | 24.1 (2.1) | 31.2 (2.0) | 20.8 (2.1) |
| Multiplicity | 7.6 (7.6) | 9.8 (9.3) | 6.8 (5.8) |
| Wilson *B* factor (Å^2^) | 35.8 | 33.0 | 24.4 |
| **Refinement** |  |  |  |
| Resolution range (Å) | 29.97-2.05 (2.10-2.05) | 29.95-1.98 (2.03-1.98) | 29.33-1.78 (1.83-1.78) |
| Completeness (%) | 99.9 (99.3) | 99.9 (99.2) | 96.8 (90.8) |
| No. of reflections | 94,164 (7,228) | 56,334 (4,345) | 76,229 (5,509) |
| *R*_work_/*R*_free_, (%) | 16.6/18.5 (24.7/26.9) | 15.0/16.6 (27.8/25.8) | 14.1/16.1 (23.9/25.6) |
| Protein chains/atoms | 4/6,692 | 2/3,302 | 2/3,359 |
| Ligand/Solvent atoms | 275/520 | 213/341 | 166/508 |
| Mean temperature factor (Å^2^) | 47.3 | 44.0 | 33.0 |
| **Coordinate Deviations** |  |  |  |
| R.m.s.d. bonds (Å) | 0.004 | 0.005 | 0.006 |
| R.m.s.d. angles (º) | 1.290 | 1.272 | 1.330 |
| **Ramachandran plot^c^** |  |  |  |
| Favored (%) | 96.0 | 98.0 | 97.0 |
| Allowed (%) | 4.0 | 2.0 | 3.0 |
| Outside allowed (%) | 0.0 | 0.0 | 0.0 |
| **Ligands** | Cap-0-RNA  SAM  Mg^2+^ | Cap-1-RNA  SAH  Mn^2+^ | Cap-1-RNA  SAH  2 Mg^2+^ |

Notes: Values in parentheses are for the outer shell. ^a^Estimated Rp.i.m. as defined by Weiss (*59*) . ^b^Pearson's correlation coefficient as defined by Karplus and Diederichs (*60*). ^c^Validation was done using MolProbity (*61*).
